# Supplementary material for: sscMap: An extensible Java application for connecting small-molecule drugs using gene-expression signatures
Source: BMC Bioinformatics. 2009 Jul 31;10:236. doi: 10.1186/1471-2105-10-236 (PMC2732627; doi:10.1186/1471-2105-10-236)
Supplement: Additional File 1 — readme. The README file of the software, which provides detailed instructions and guided tours for running the program. [file 1471-2105-10-236-S1.pdf]

sscMap: Connecting small-molecule drugs using gene-expression signatures

The sscMap program implements the method introduced in [1] to test the connections between Gene Signatures and Reference Gene-Expression Profiles. It may be helpful to read some related papers [1,2] first to learn more about the methodology itself. This document can be regarded as a supplementary to the paper that introduces the sscMap program [3].

The sscMap program can be run in two execution modes: as a command line program, or as a GUI (Graphical User Interface) application. The instructions for running the program as a command line can be found near the end of this document, while the two tours below will guide you through the GUI mode. You should have Java 1.6 (or later version) installed on your computer to run the program. By going through the two guided tours, users should be able to get a fairly good grasp of how the program works. After that you can try to run queries using your own gene signatures.

The sscMap software is bundled with a default collection of 6100 reference gene-expression profiles based on the Broad Institute Connectivity Map 02 dataset (http://www.broad.mit.edu/cmap/). To query this default collection of reference profiles using your own gene signature(s), you need first prepare the query files in the format as those examples in the folder "queries", one file for each gene signature. The files should be tab-delimited text, and gene IDs should be represented by Affymetrix HG-U133A probe-set IDs, as these are the IDs used in the default reference profiles. If your gene IDs are not already Affymetrix HG-U133A probe-set IDs, please use the corresponding Affymetrix annotation files to map them to these IDs.

The sscMap software can be extended by adding custom collections of reference profiles. Tour 2 actually uses a small example of custom extension. The section after Tour 2 gives a detailed description of the general contracts for adding a custom collection of reference profiles to sscMap.

References:

[1] Shu-Dong Zhang and Timothy W. Gant, A simple and robust method for connecting small-molecule drugs using gene-expression signatures, BMC Bioinformatics 2008, 9:258. DOI:10.1186/1471-2105-9-258.
Highly accessed (A highly accessed article on the BMC Bioinformatics website).

[2] J Lamb J et al, The Connectivity Map: Using Gene-Expression Signatures to Connect Small Molecules, Genes, and Disease. Science 2006, 313(5795):1929-1935.

[3] Shu-Dong Zhang and Timothy W. Gant, sscMap: An extensible Java application for connecting small-molecule drugs using gene-expression signatures.

Tour 1: Querying the bundled collection of reference profiles with 5 gene signatures

(1) Launch the program

Go to the program’s main folder “sscMap”; double click the “sscmap-gui.bat” file to launch the program. If you are using Unix or Linux, you may need first add execution permission to the file "sscmap-gui", and then start the program. The commands you need to type in are:

```
chmod +x sscmap-gui
sscmap-gui
```

A Graphical User Interface similar to the one shown below should appear.

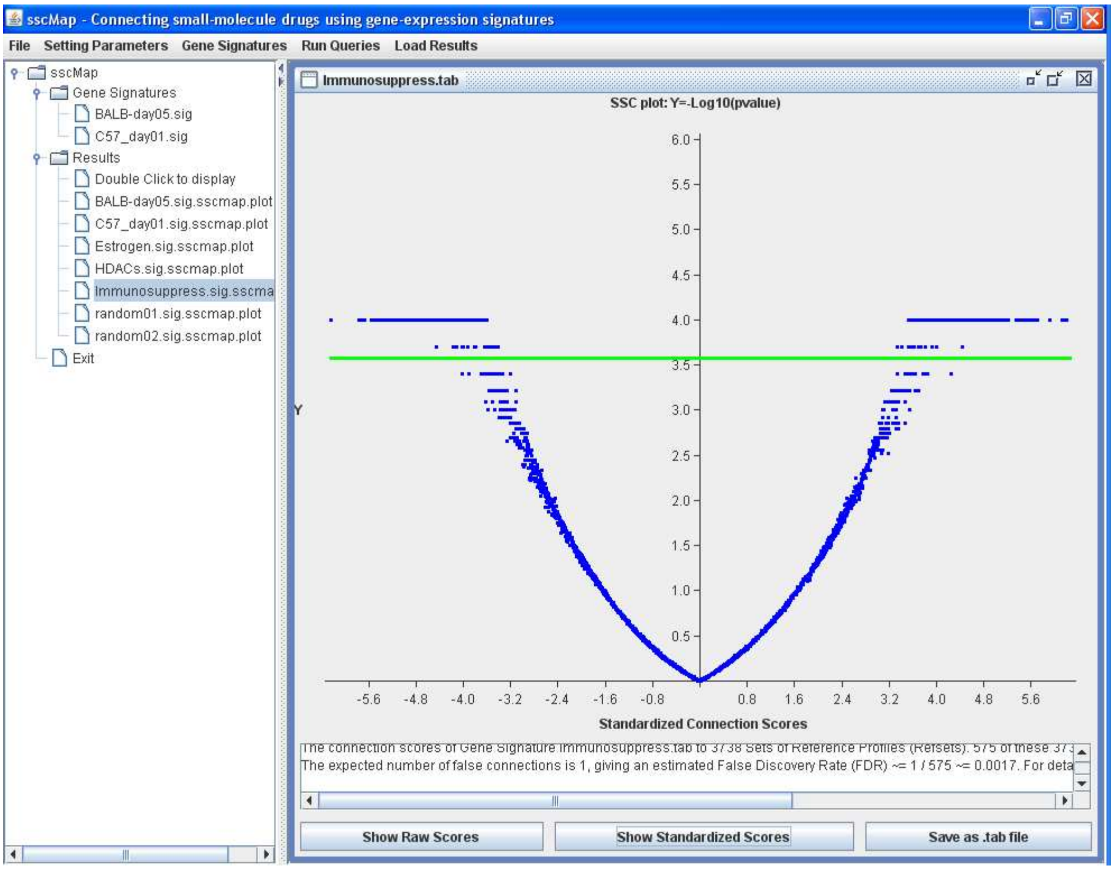

(2) Clearing up for a fresh start

Click the “Gene Signatures” menu on the menu bar, and then select the “Remove All Gene Signatures” item, to clear up the Gene Signature list from previous run.

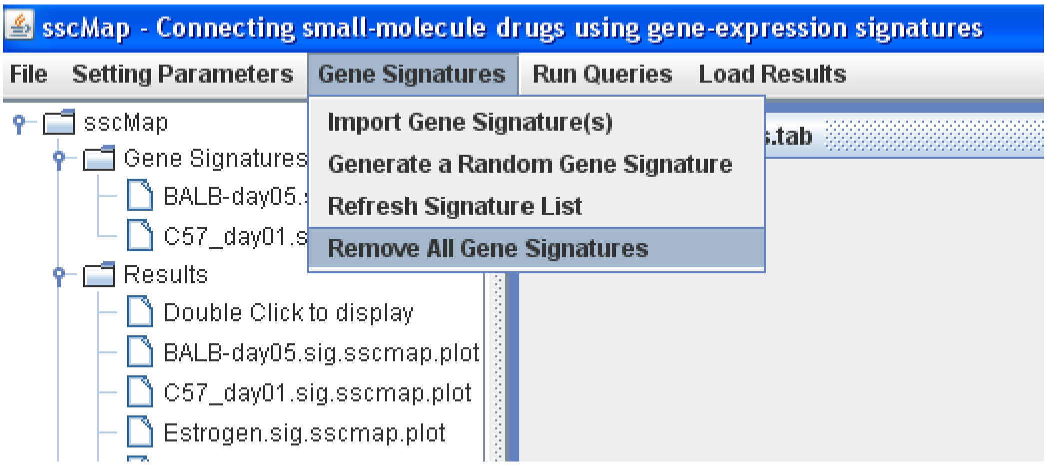

Click the "Load Results" menu on the menu bar, then select "Remove All Result Nodes" to clear up the results nodes.

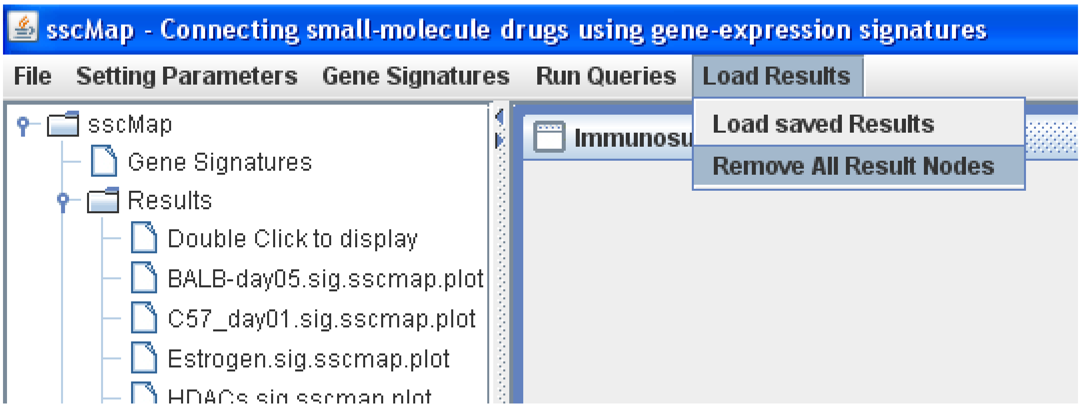

After clearing up, the GUI looks like shown below.

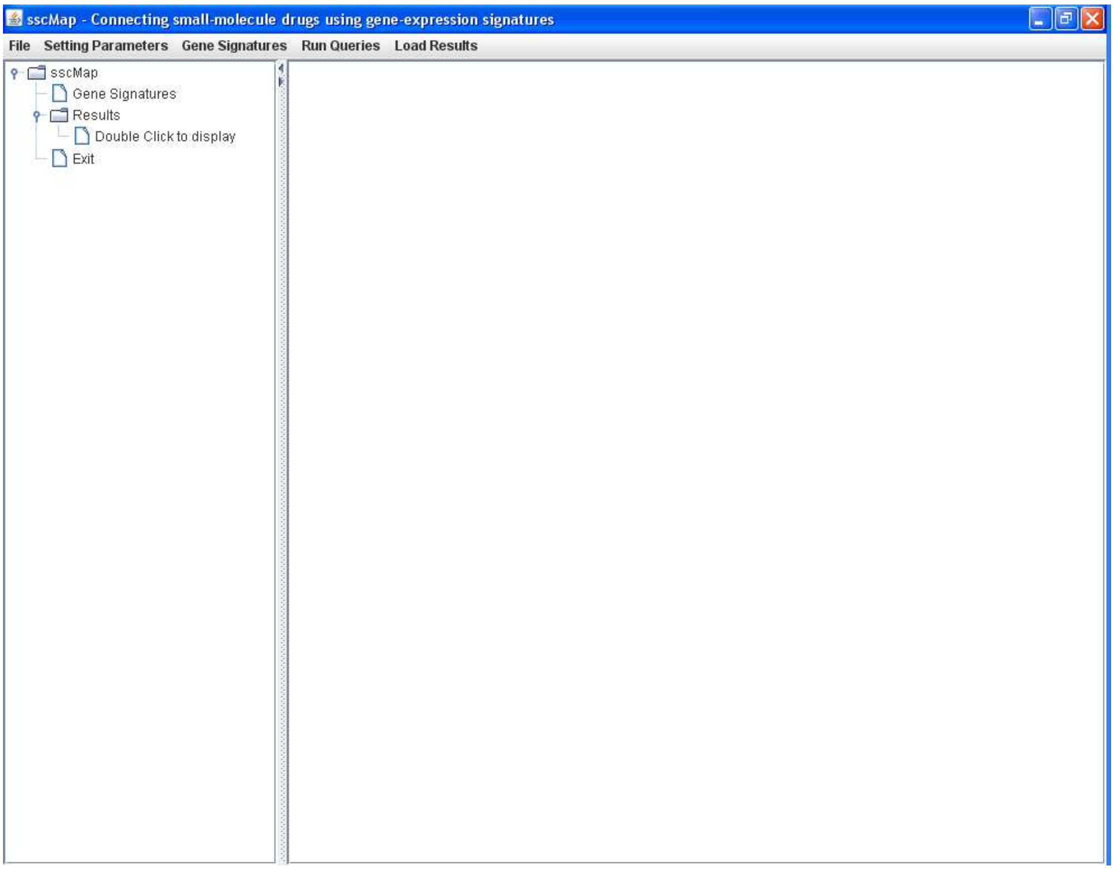

(3) Loading default settings

Click the menu "Setting Parameters" on the menu bar, and then select the "Load Default Settings" item.

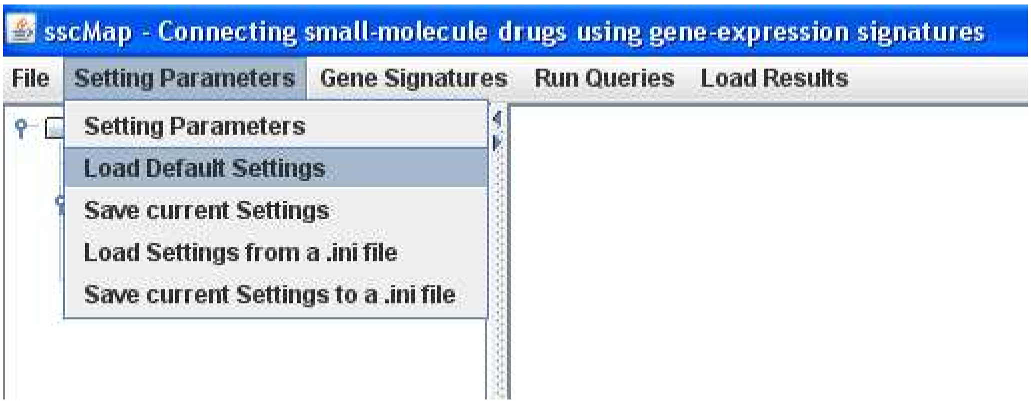

This will bring up the "sscMap Settings" window, showing the current settings (The default settings in this case).

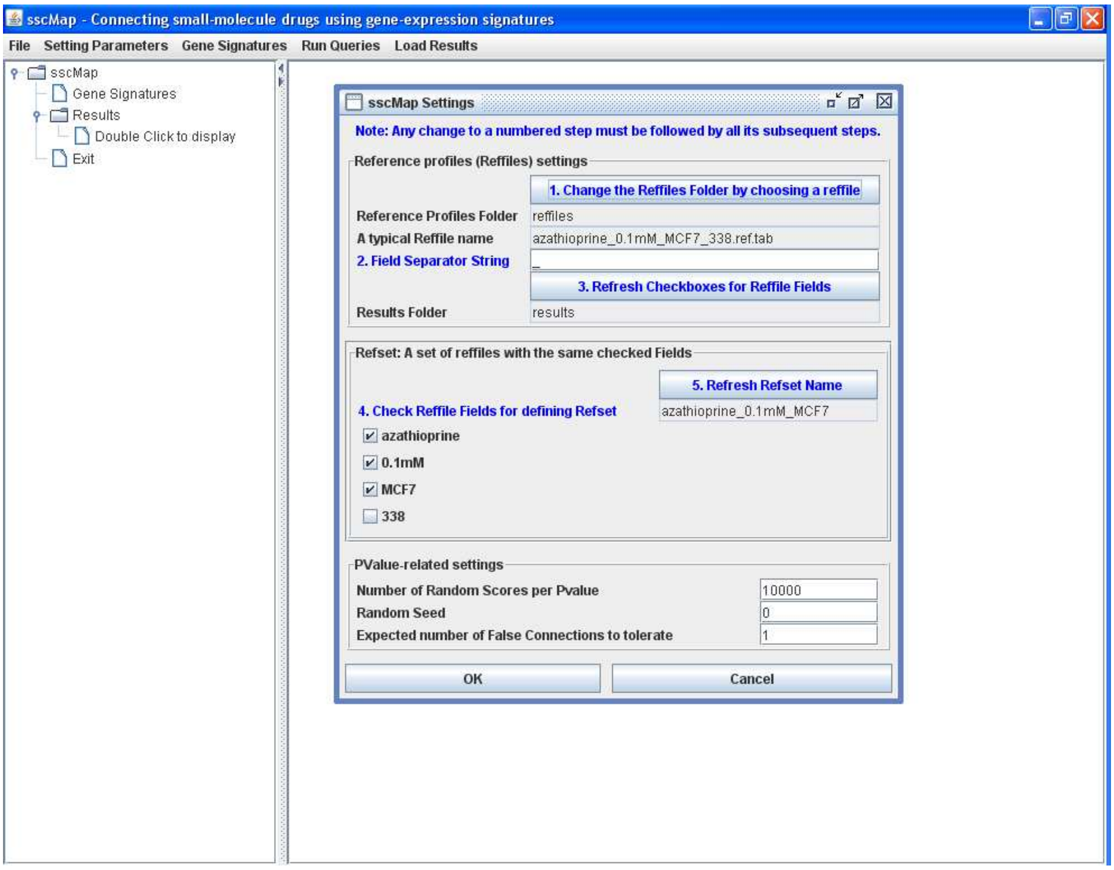

As we are going to use the default settings in this case, no need to change anything, just press the “OK” button to close the window.

(4) Load the gene signatures

Click the “Gene Signatures” menu on the menu bar; select the item “Import Gene Signature(s)”.

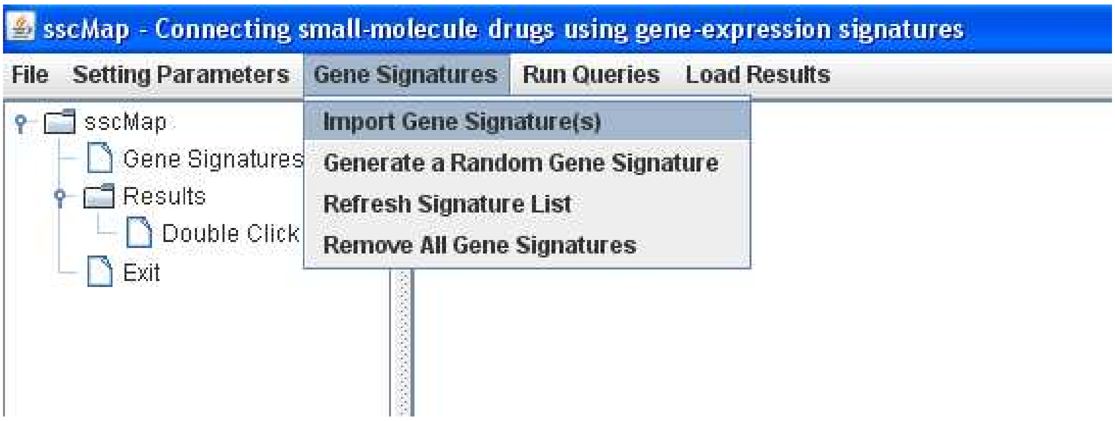

Browse to the folder “queries” under the program main folder “sscMap”, select the five Gene Signature files there to load them into the gene signature list (Multiple selection allowed). These five Gene Signature files are: “Estrogen.sig”, “HDACs.sig”, “Immunosuppress.sig”, “random01.sig”, and “random02.sig”.

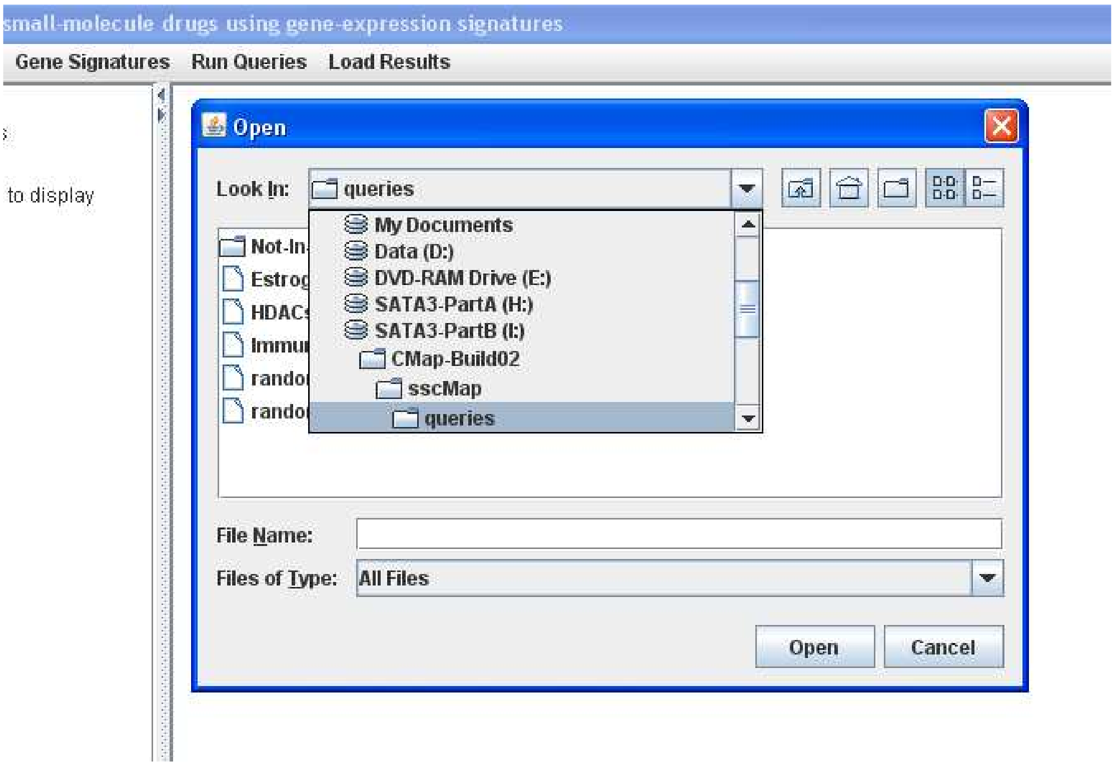

And they will appear as five nodes under the “Gene Signatures” node, as shown below.

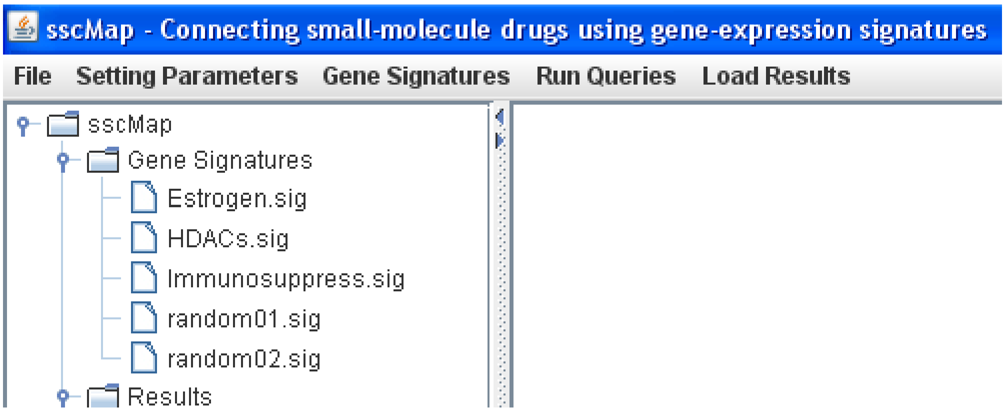

Now we are ready to run the queries.

(5) Run the queries

Click the “Run Queries” menu on the menu bar, and select “Run queries with current settings”.

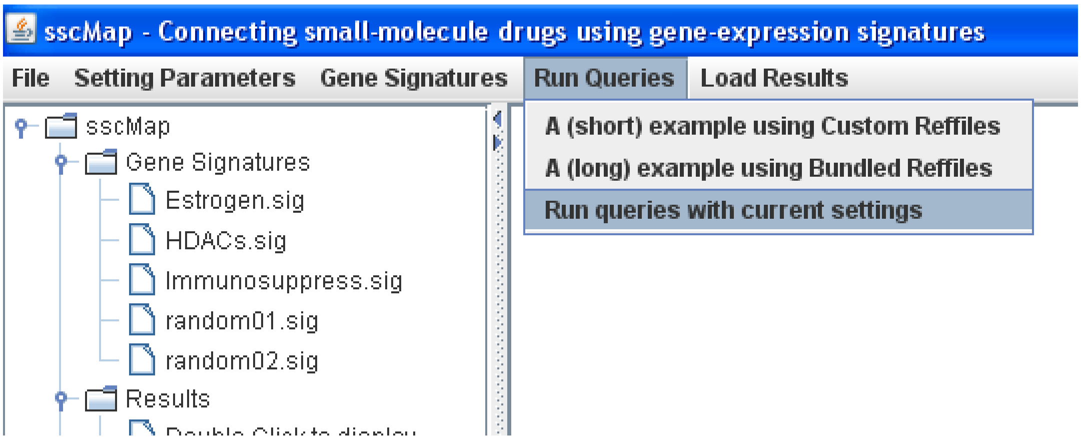

A progress monitor window will pop up shortly indicating the progress being made. In this example, as shown below, the program is calculating 18690 connection scores and estimating 18690 p-values. It takes a couple of hours to finish on a typical today's desktop computer, as the estimation of p-values is the most time-consuming part of the calculation (2 hours and 7 minutes on my laptop computer with an Intel Core Duo T2300E / 1.66 GHz processor; 1 hour and 26 minutes on my desktop computer with an AMD Athlon 64 X2 6000+ AM2 3.0GHz processor).

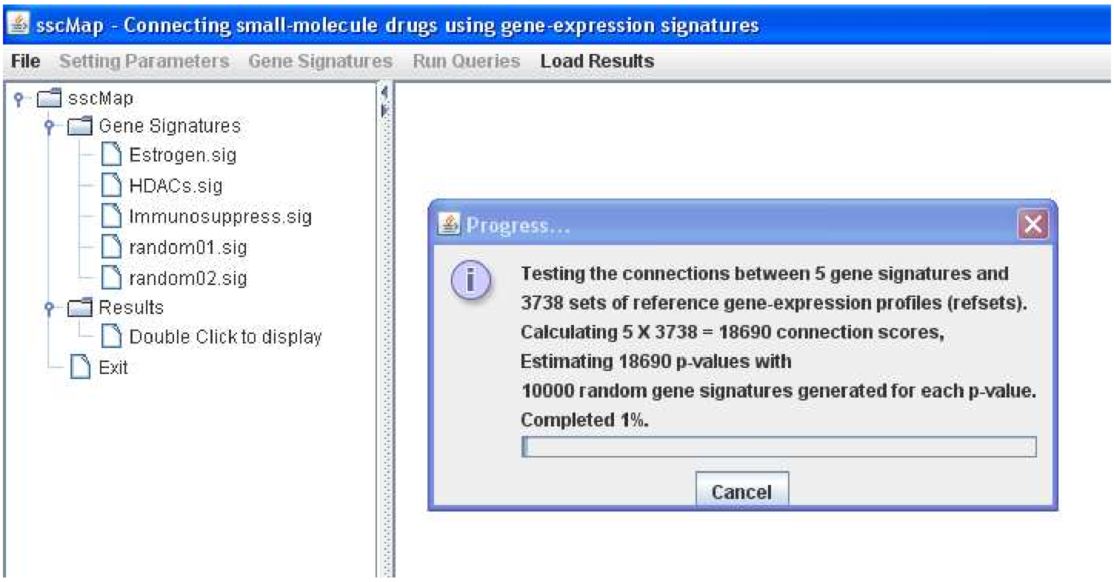

Once the calculation is completed, the "Results" node will be populated with five result nodes, one for each gene signature in the Gene Signature list. A graph showing the connection scores and p-values will be displayed on the right. The caption under the graph summarizes the results shown in the graph.

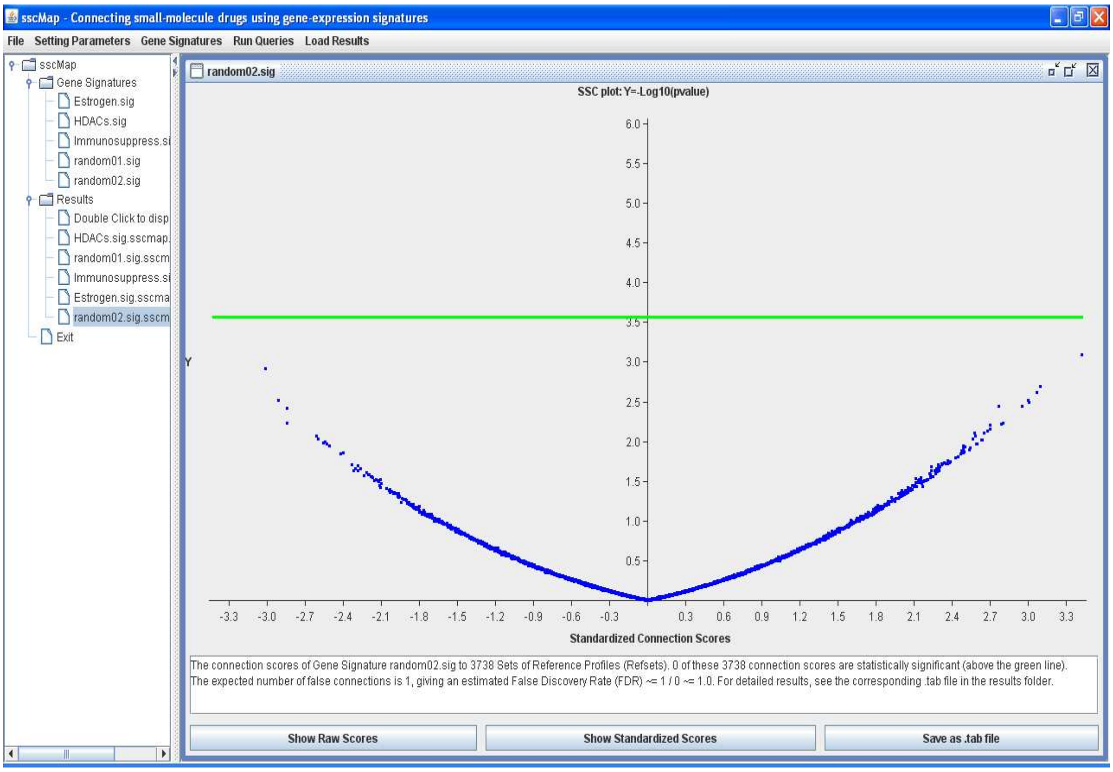

Pointing the mouse to a data point on the graph and pressing a mouse button will bring up a small window displaying the detailed information for that data point. When the mouse button is released, the information window will disappear.

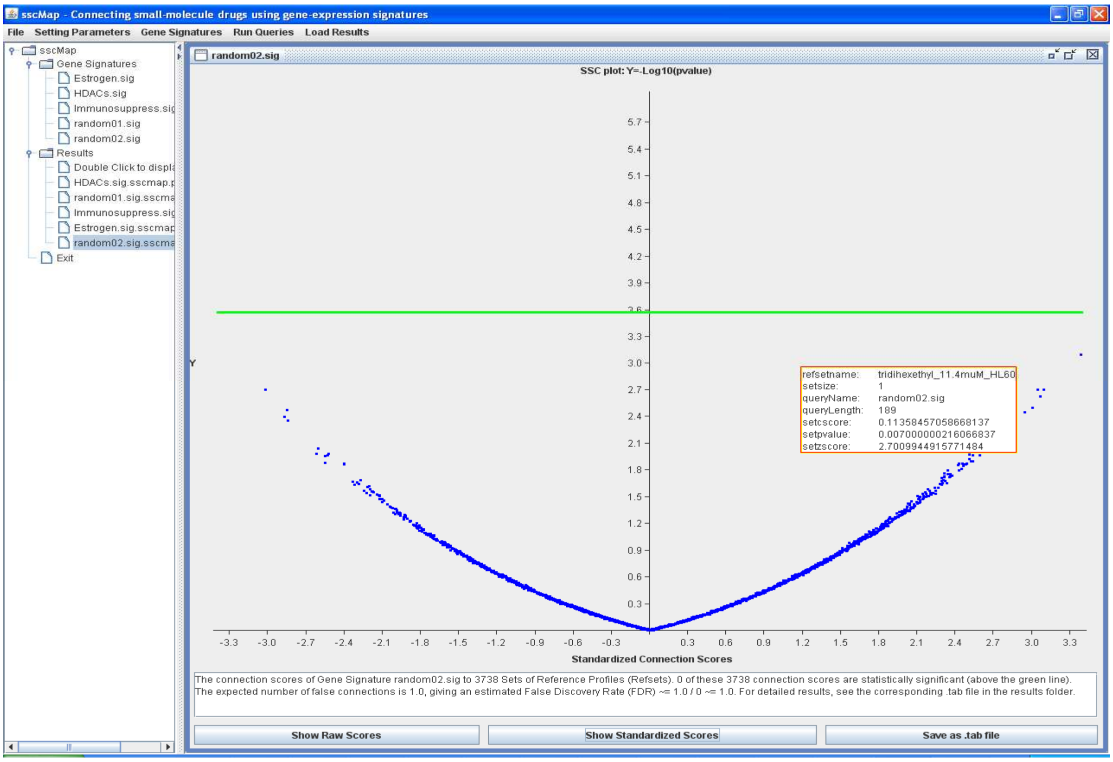

(6) Viewing and interpreting the results

Let's take the HDACs gene signature as an example. Double clicking the result node "HDACs.sig.sscmap.plot", a graph for that node will be displayed on the right as below. The horizontal axis is for connection scores and the vertical axis is $Y= -\log_{10}(\text{pvalue})$. So if a data point on the plot has a Y-coordinate around 3, you know that the original p-value is of the order 10^{-3}. The green horizontal line on the graph indicates where the threshold p-value is set. Any connection score with a p-value less than that threshold (any data point above the green line on the graph) is considered as statistically significant. The threshold p-value is in fact set as

alpha = N_falses / N_sets = 1 / 3738 ~= 0.00027,

where N_false=1 is the number of false connections the user is willing to tolerate for each gene signature, and N_sets=3738 is the number of Refsets being queried in this run. Thus the number of connection scores obtained for each gene signature is also N_sets. By setting the threshold p-value as such, we should expect that on average there will be N_falses=1 false connections among the significant connection scores. The expected number of false connections to tolerate (N_falses) can be viewed and/or changed from the "sscMap Settings" window when the "Setting Parameters" menu item is selected. Its default value is 1.

In the graph shown below, you may notice that the Y-coordinates of the data points become flat ("saturate") at around Y=4.0, indicating that the smallest p-values are of the order 10^{-4} for the data shown here. This is because the number of random gene signatures generated for each p-value estimation was 10000. So the lower limit of p-values that can be estimated is of the order 10^{-4}. The true p-values of those saturated data points are likely to be much smaller. But for the purpose of identifying significant connections using the current threshold (alpha=0.00027, the green line), this limit (10^{-4}) for p-value estimation is adequate. Increasing the number of random gene signatures for each p-value estimation to 100000, for example, will require much longer computational time. The set of significant connections identified by the longer run, however, is not much different. So overall, using 10000 random gene signatures to estimate each p-value is probably the right balance to strike.

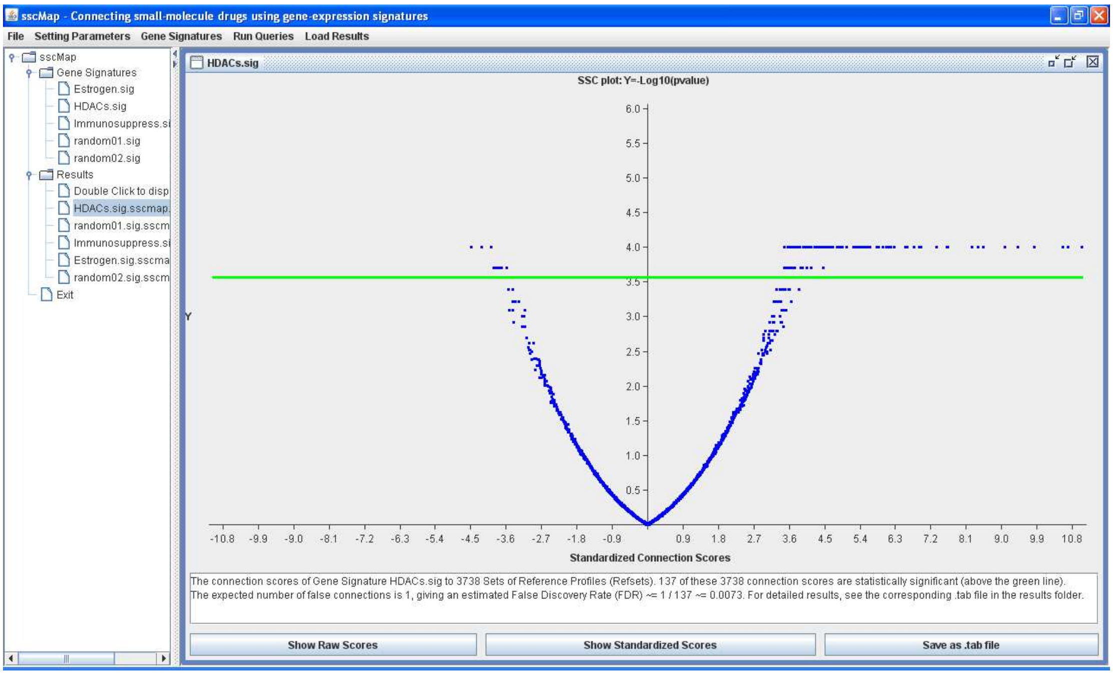

There are three buttons at the bottom of the graph. Pressing the Button “Show Raw Scores” will show the original connection scores defined by Equation (6) in [1], and pressing the “Show Standardized Scores” button will show the standardized connection scores. The standardized connection score is just the original connection score normalized (divided) by the sample standard deviation of the random scores generated during the p-value estimation.

The “Save as .tab file” button allows users to export the results and save them to a tab-delimited text file, which can be opened and viewed using MS-Excel or other similar spreadsheet software.

In fact, two files were already saved in the “Results” folder for each gene signature when the calculation was completed. One is a “*.sscmap.tab” file, in tab-delimited text format, which basically lists the connection results of the gene signature to all the Refsets queried. This “*.sscmap.tab” file can be opened and viewed with MS-Excel or other similar spreadsheet software. It is the main result file for users to take away and to work on.

The other file “*.sscmap.plot” is in binary format, and is only be to read by the current sscMap program. The “Load Saved Results” item under the “Load Results” menu allows users to load previously saved “*.sscmap.plot” files and display them in graph similar to the one we have seen above.

(7) Exit the program

Double click the “Exit” node to exit the program, or Click “File” on the menu bar, and select “Quit”.

Tour 2: Querying a custom collection of reference profiles with 2 gene signatures.

(1) Launch the program

Go to the program's main folder "sscMap"; double click the "sscmap-gui.bat" file to launch the program. If you are using Unix or Linux, you may need first add execution permission to the file "sscmap-gui", and then start the program. The commands you need to type in are:

```
chmod +x sscmap-gui
sscmap-gui
```

A Graphical User Interface (GUI) similar to the one below should appear.

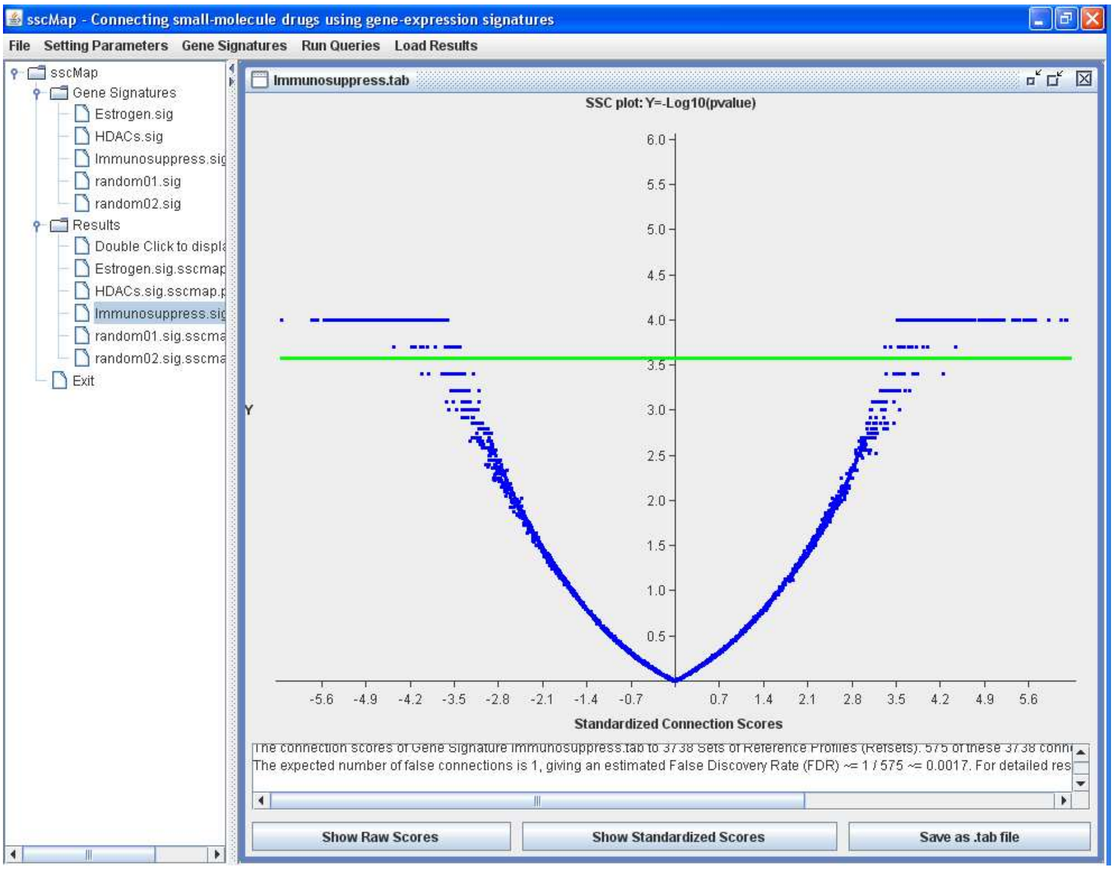

(2) Clearing up for a fresh start

Click the "Gene Signatures" menu on the menu bar, and then select the "Remove All Gene Signatures" item, to clear up the Gene Signature list from previous run.

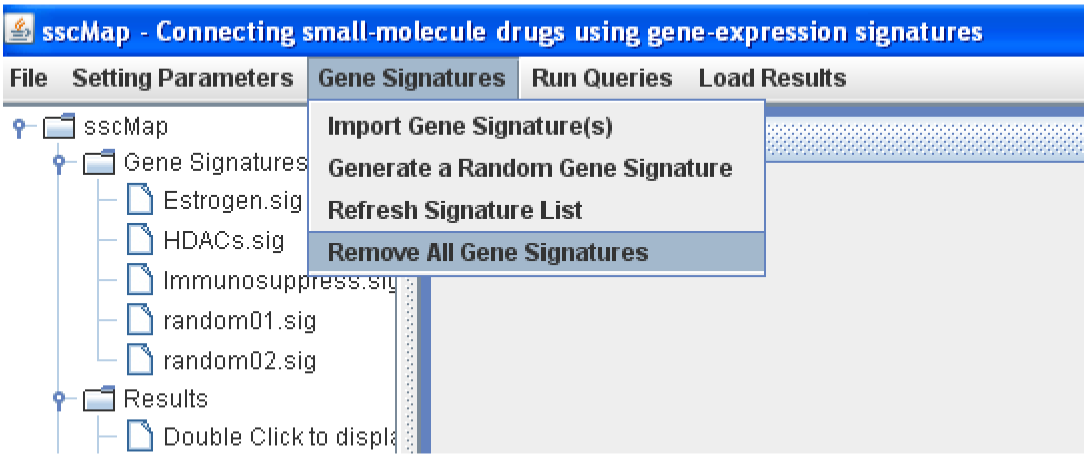

Click the “Load Results” menu on the menu bar, then select “Remove All Result Nodes” to clear up the results nodes.

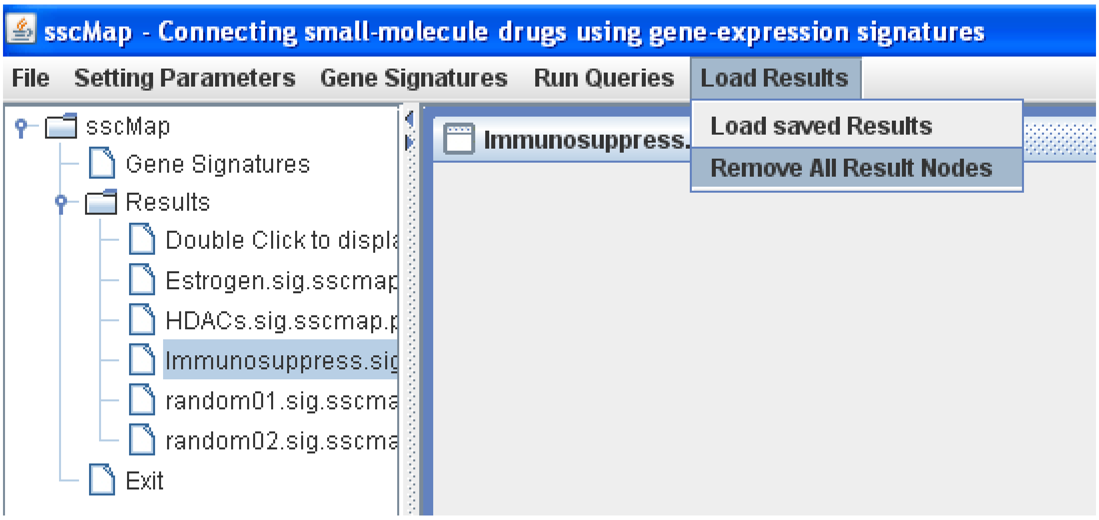

(3) Setting essential parameters

Click the “Setting Parameters” menu on the menu bar, then select “Setting Parameters” to bring up the “sscMap Settings” window.

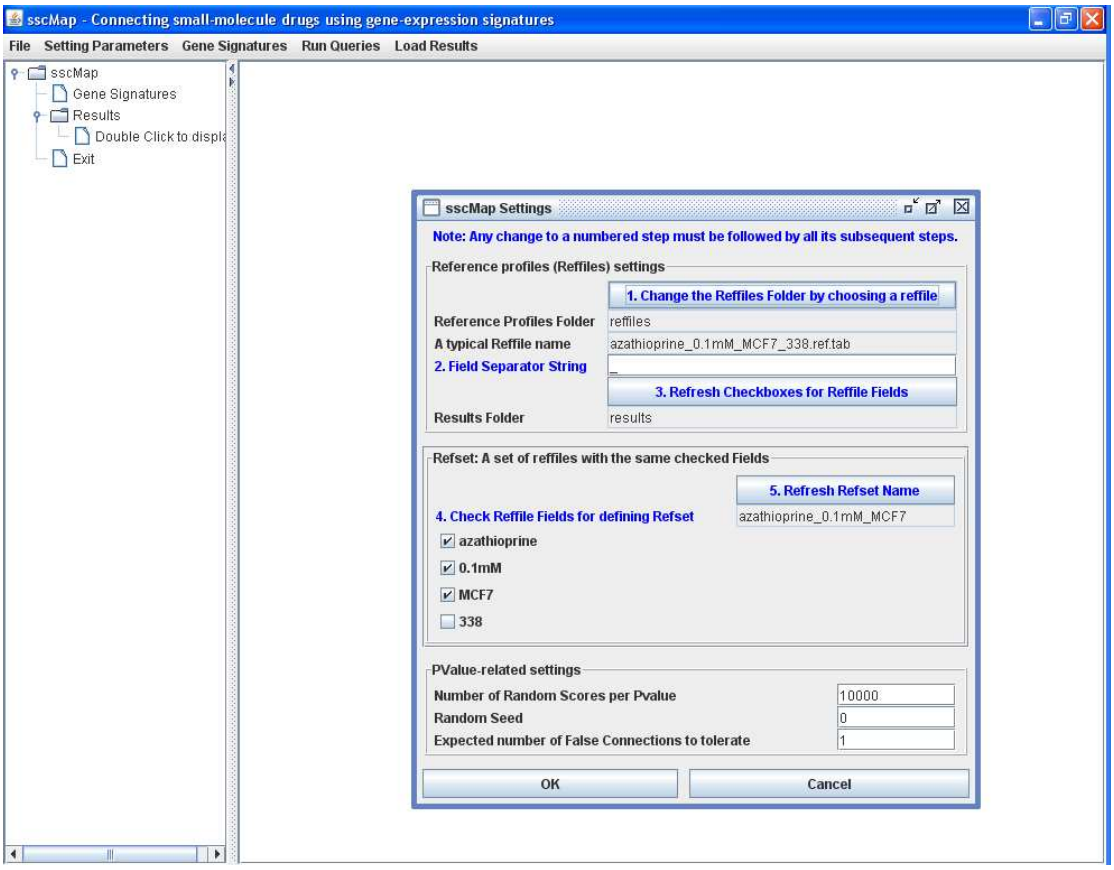

In this example, we are going to use a custom collection of reference profiles stored in a subfolder under “custom-example”.

Click the Button “1. Chang the Reffiles Folder by choosing a reffile”, and browse to the folder “custom-example/custom-reffiles” under the program’s main folder “sscMap”.

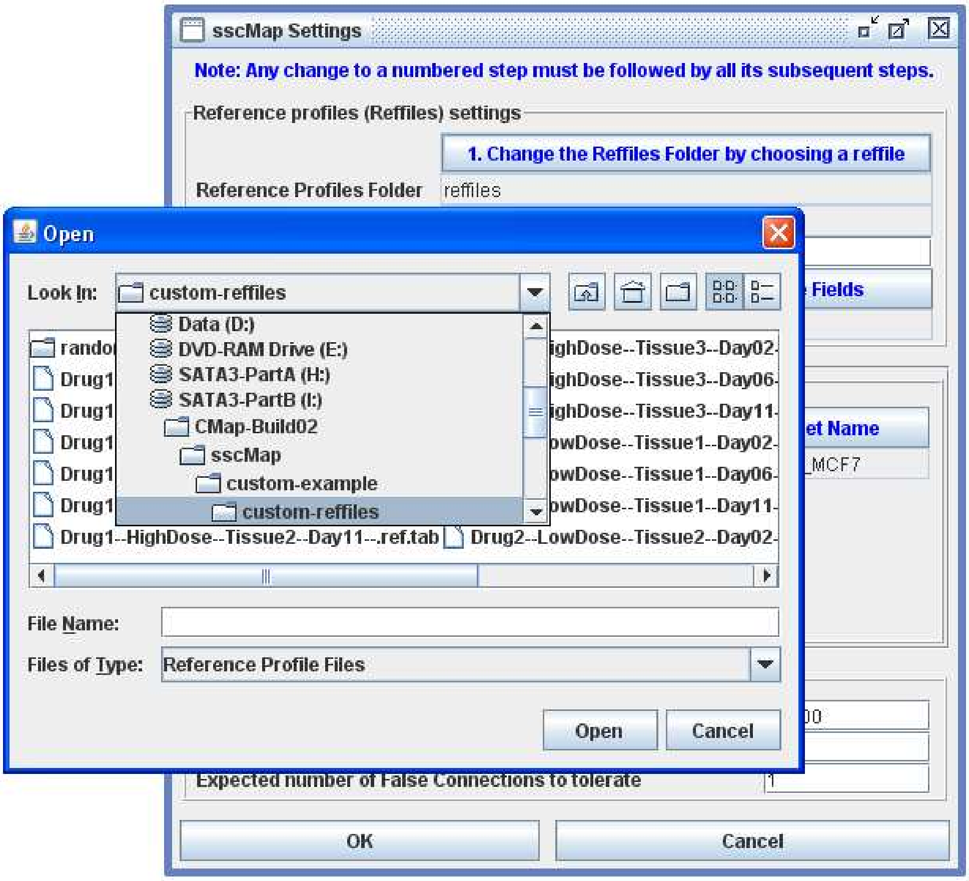

Select the file "Drug1--HighDose--Tissue1--Day02.ref.tab" (or any other reffile), then press the "Open" button. As you can see, the Reference Profiles Folder is now "….\sscMap\custom-example\custom-reffiles", and a typical reffile name is "Drug1--HighDose--Tissue1--Day02.ref.tab".

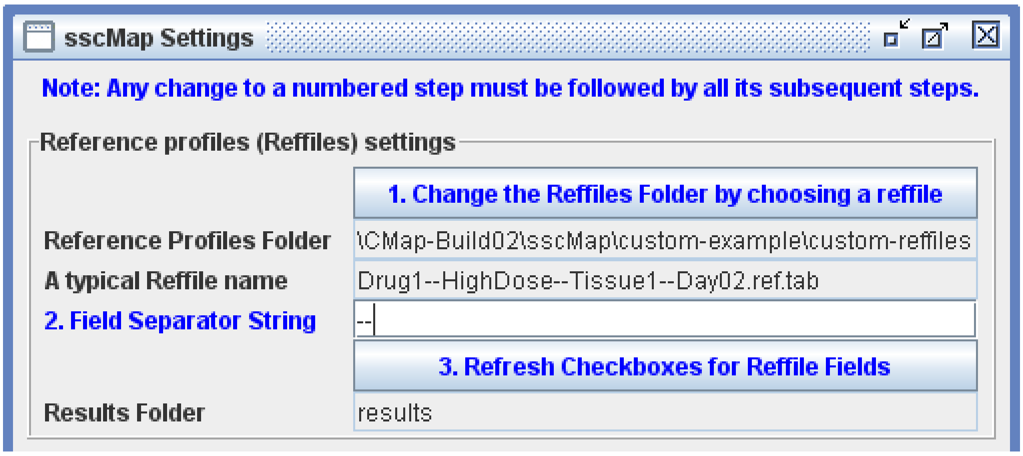

The naming of reffiles in this folder apparently suggests that the string -- is to be used as a Field Separator to divide a reffile name into several Fields. In this example, these Name Fields are: drug name, dose, tissue type, and time point.

So we type in the Field Separator String -- (two hyphens) into the text box.

Click the Button “3. Refresh Checkboxes for Reffile Fields”, the checkboxes will be updated as below.

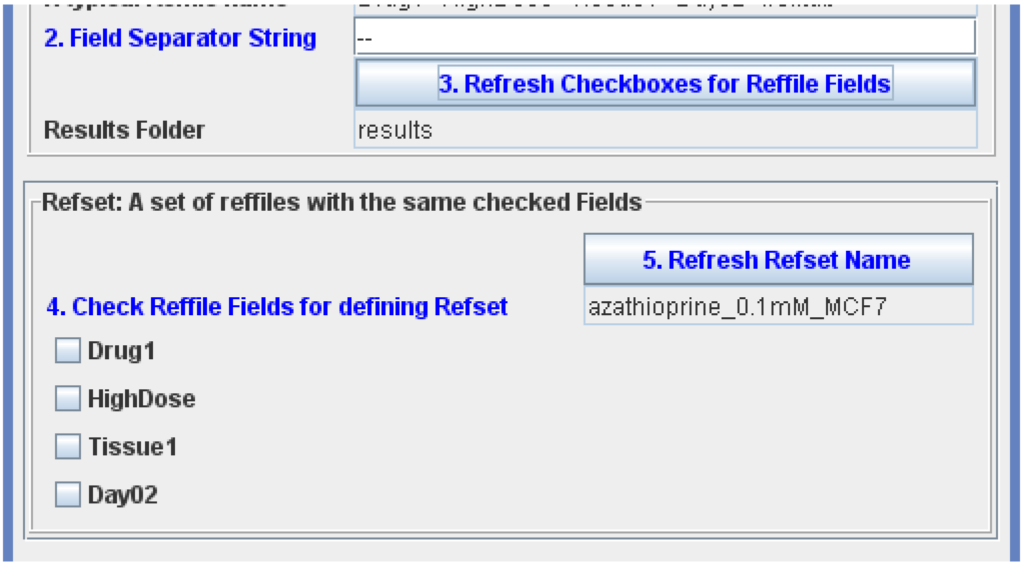

Now we must decide which name fields to use for defining a Refset. A Refset is defined as a set of reffiles with the same selected Name Fields. In this example, we are going to use the drug name and tissue type to define a refset. This means that all reference profiles with the same drug and tissue type will be taken as forming a refset, disregarding the dose and time point.

So we check the two Name Fields (Drug and Tissue) then click the Button “5. Refresh Refset Name”.

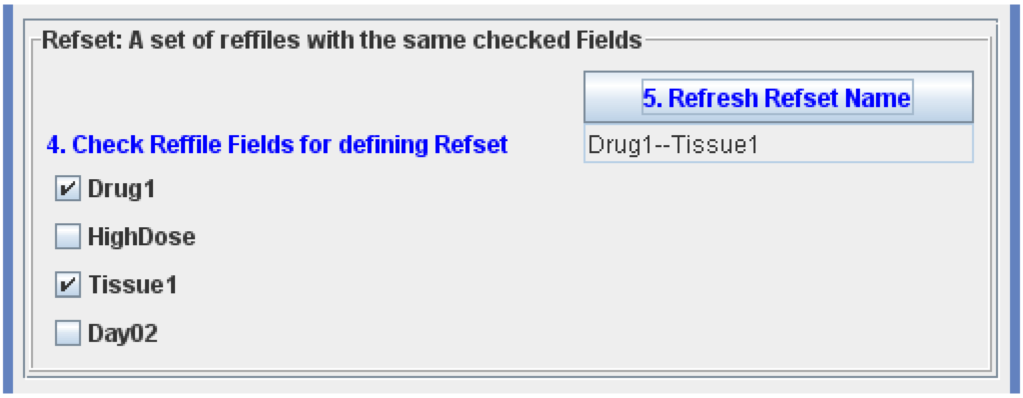

We leave the pvalue-related settings at their default values, and click to “OK” button to close the “sscMap Settings” window.

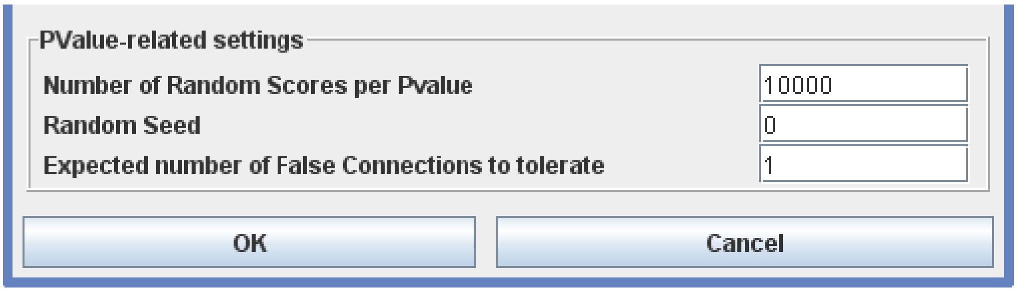

(4) Load the gene signatures

Click the “Gene Signatures” menu on the menu bar, select “Import Gene Signature(s)”.

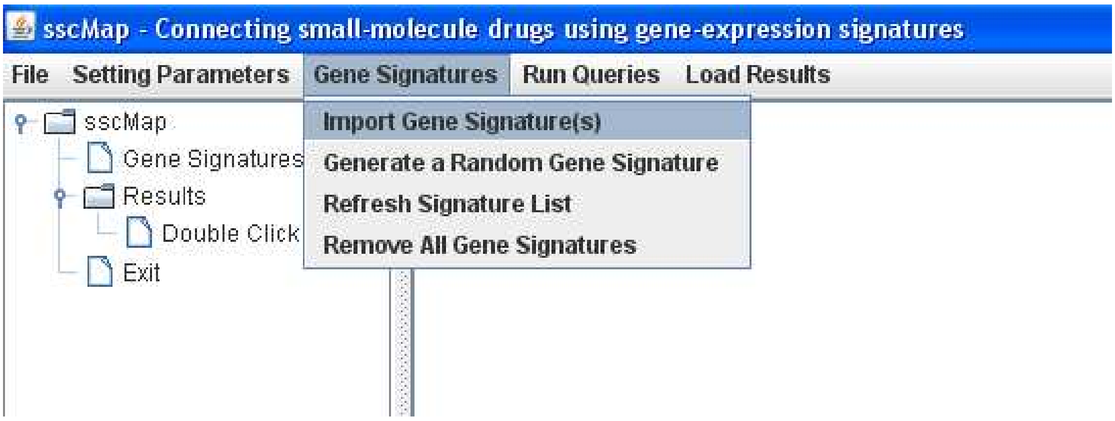

Browse to the folder “custom-example/queries” under the program main folder “sscMap”, select the two Gene Signature files (“BALB-day05.sig” and “C57_day01.sig”) there to load them into the gene signature list.

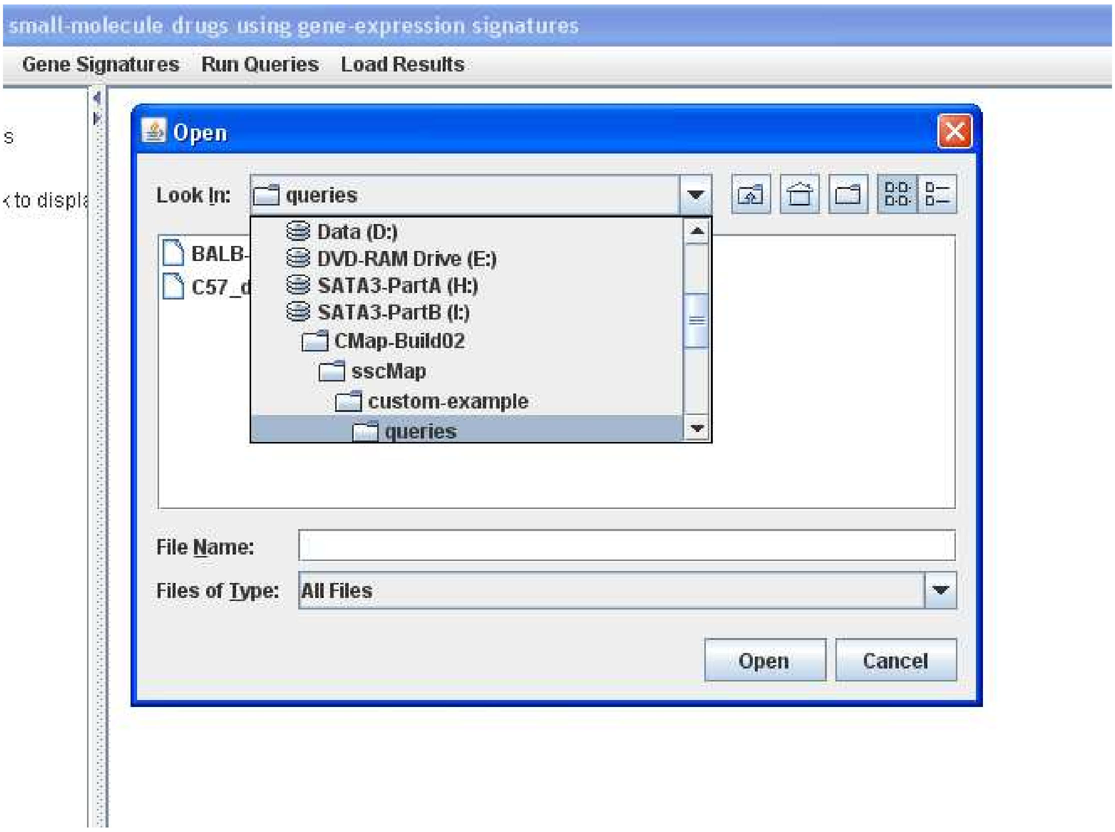

And they will appear as two nodes under the "Gene Signatures" node, as shown below.

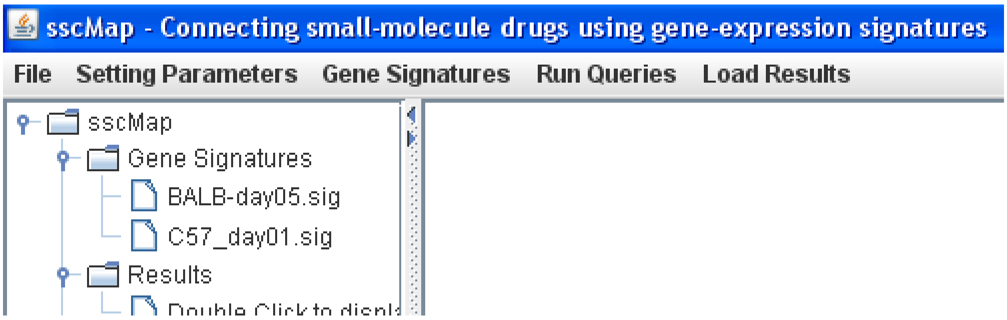

Now we are ready to run the queries.

(5) Run the queries

Click the "Run Queries" menu on the menu bar, and select "Run queries with current settings".

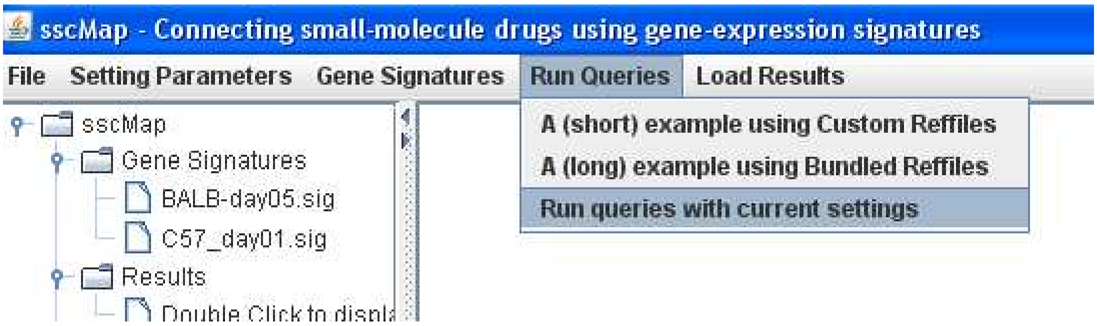

A progress monitor window will pop up shortly indicating the progress made.

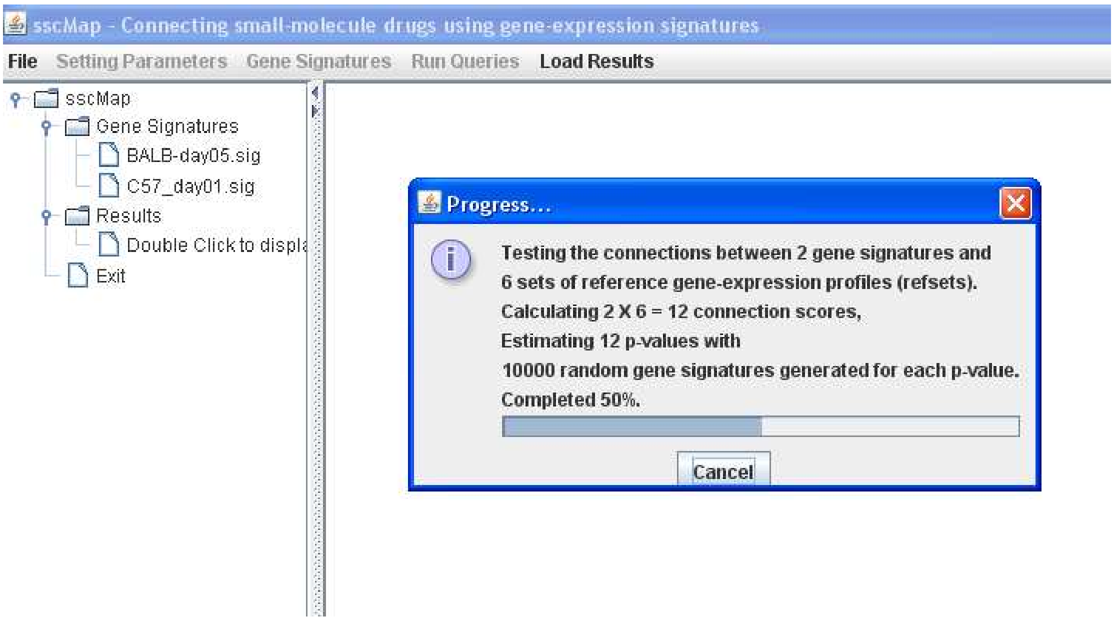

Once the calculation is completed, the "Results" node will be populated with two result nodes, one for each gene signature in the Gene Signature list. A graph showing the connection scores and p-values will be displayed on the right. The caption under the graph summarizes the results shown in the graph. Double clicking any result node will display the graph for that node.

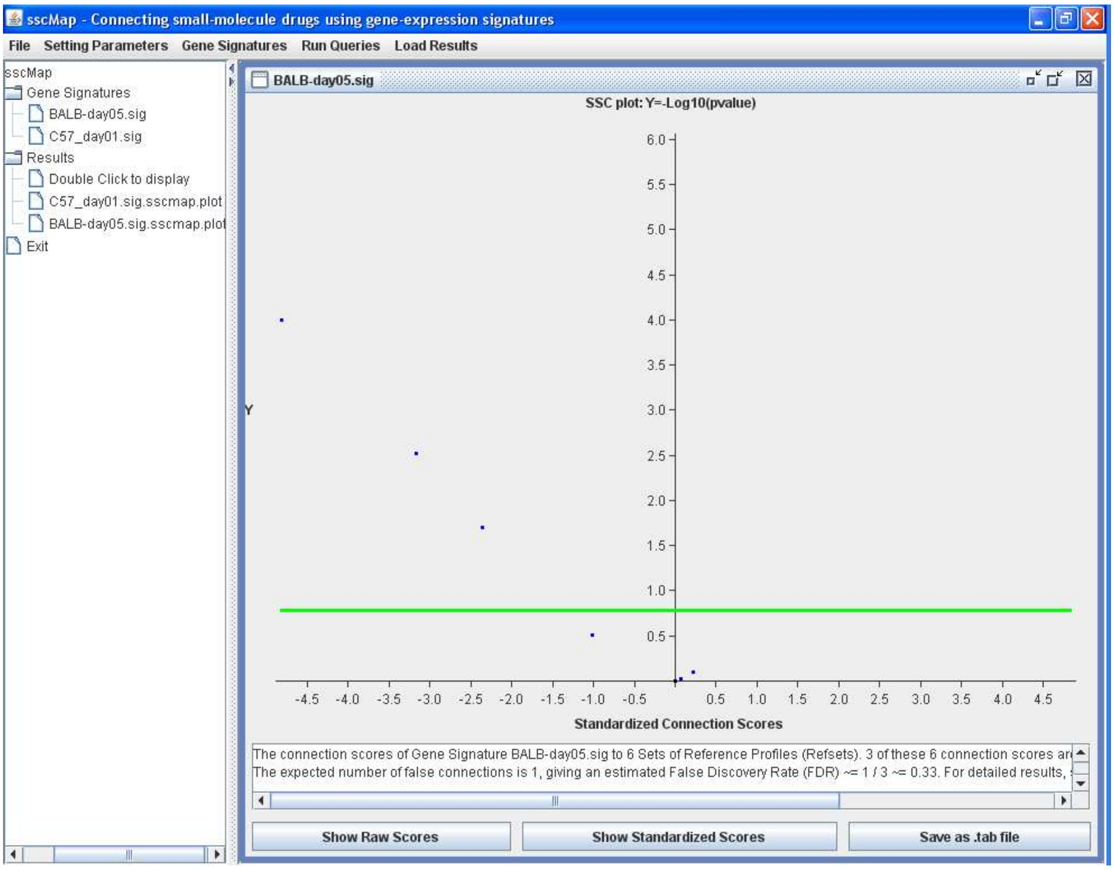

(6) Exit the program

Double click the "Exit" node to exit the program, or Click "File" on the menu bar, and select "Quit".

Adding custom reference profiles to sscMap

As an example, we have included with the sscMap program a folder called *custom-example*, which contains all the key components of a custom extension to the application. Following the example provided users should be able to build their own extension.

This section describes the general contracts for a custom collection of reference profiles to be added to sscMap. A custom ref-files directory must contain the following items:

1. A file called *IDstore.tab*;
2. A number of ref-files with extension name **.ref.tab*.

The *IDstore.tab* file is essential, as it lists all the gene IDs present in these ref-files. Each ID is a unique and case-insensitive string of characters, e.g. a probe-set ID in an Affymetrix microarray platform. *IDstore.tab* is a text file with only one column, the first row being the column name; from the second row to the last row are the gene IDs,

one ID per row. The IDs in the *IDstore.tab* file are not required to be in any particular order, as they will be sorted alphabetically anyway during the program's execution.

A ref-file basically specifies the ranks of all the IDs defined in *IDstore.tab*, and thus contains the same number of IDs. A ref-file is also a tab-delimited text file, with the first row giving the column names; the first column is for gene IDs and the second column for their corresponding singed ranks. As described in [1], the most important gene ID is assigned the highest rank N if it is up-regulated, or -N for down-regulation, where N is the total number of gene IDs defined in *IDstore.tab*. A ref-file may contain extra columns to provide more information such as the expression ratio, log-ratio, raw expression values etc, but only the first two columns are used by the program, and the other columns are ignored. For an example of a ref-file with extra columns, see the file “custom-example/custom-reffiles/Drug2--LowDose--Tissue3--Day11.ref.tab”.

Notice that in the default collection of ref-files, the gene IDs are those defined by the Affymetrix probe-set IDs of microarray platform HG-133A, while in the example of custom reference profiles, the gene IDs are those defined by the Affymetrix microarray platform Rat230_2. This shows that users have the freedom to use whatever gene IDs in their own *IDstore.tab* file, the only requirement being that there should be no duplications and the ID strings are case-insensitive. Needless to say, when you want to query a collection of reference profiles, whether it is the bundled collection or a custom collection, the query gene signatures should always use gene IDs defined by the *IDstore.tab* corresponding to the ref-files to be queried.

Instructions for running sscMap as a command line

To run the program using the example queries already in the folder "queries",
(1) Open an example query using MS Excel as a tab-delimited text file, to get familiar with the format of query files.

(2) Go to the program main folder; double click the file "run-sscmap.bat" to start the program. If you are using Unix or Linux, you may need first add execution permission to the file "run-sscmap", and then start the program. The commands you need to type are:

```
chmod +x run-sscmap
run-sscmap
```

(3) After the program ends, go to the "results" folder, open the corresponding "*.sscmap.tab" files with Excel to view the results.

To query sscMap with your own query gene signature(s),
First, prepare the query signature files in the format as those examples in the folder "queries", one file for each gene signature. The files should be tab-delimited text, and gene IDs should be represented by Affymetrix HG-U133A probe-set IDs.

Second, put all your query gene signatures into the folder "queries".

Third, edit the "parameters.ini" file to set the input parameters accordingly, e.g., whether your query signatures are ordered or un-ordered gene list. The "parameters.ini" file is a plain text file, and can be edited with any text editors.

Finally, double click the run-sscmap.bat file to run the program. Or preferably, launch a MS-DOS window first, then change the working directory to the main program folder, and type the command "run-sscmap" to start the program.

The results will be saved in the "results" folder as tab-delimited text files with name "*.sscmap.tab", which can be opened and viewed using a spreadsheet software such as MS Excel.
